# Assessing the Surrogate Susceptibility of Oxacillin and Cefoxitin for Commonly Utilized Parenteral Agents against Methicillin-Susceptible *Staphylococcus aureus*: Focus on Ceftriaxone Discordance between Predictive Susceptibility and *in Vivo* Exposures

**DOI:** 10.3390/pathogens4030599

**Published:** 2015-07-30

**Authors:** Nayon Kang, Seth T. Housman, David P. Nicolau

**Affiliations:** 1 Department of Pharmacy Services, Hartford Hospital, 80 Seymour Street, Hartford, CT 06102, USA; E-Mail: nykang2@uky.edu; 2 Center for Anti-Infective Research and Development, Hartford Hospital, 80 Seymour Street, Hartford, CT 06102, USA; E-Mail: seth.housman@wne.edu; 3 Division of Infectious Diseases, Hartford Hospital, 80 Seymour Street, Hartford, CT 06102, USA

**Keywords:** methicillin-susceptible *S. aureus*, susceptibility, surrogate

## Abstract

Susceptibility testing with the use of surrogate agents is common among clinical microbiology laboratories. One such example is oxacillin and cefoxitin for β-lactams against methicillin-susceptible *Staphylococcus aureus* (MSSA). This study aimed to assess the surrogate predictive value (SPV) of oxacillin and cefoxitin for the susceptibility of commonly utilized parenteral β-lactams against MSSA as well as to evaluate the concordance between predictive susceptibility testing and the *in vivo* exposures for ceftriaxone. Broth microdilution MICs were determined for cefazolin, cefoxitin, ceftaroline, ceftriaxone, nafcillin, and oxacillin against a national collection of 1238 MSSA from US hospitals. Pharmacodynamic profiling was utilized to establish a clinical breakpoint for commonly utilized doses of ceftriaxone. Oxacillin had good SPVs for all the β-lactams tested, whereas cefoxitin produced unacceptable major errors for all four agents and thus appears to be an unacceptable susceptibility surrogate. While oxacillin is an adequate surrogate based on the currently defined laboratory criteria, our data also suggest that caution should be exercised when incorporating this testing approach in the clinical setting in view of the fact that the MIC distribution of MSSA coupled with the commonly utilized low doses of ceftriaxone may result in inadequate *in vivo* exposures against this pathogen.

## 1. Introduction

For decades, the parenteral antistaphylococcal penicillins, oxacillin and nafcillin, have been utilized for the treatment of methicillin-susceptible *Staphylococcus aureus* (MSSA) infections [1]. Alternatively the cephalosporins have been advocated as a treatment option for MSSA given their convenience in dosing and tolerable side effect profile [2]. Based on the pharmacologic properties of ceftriaxone it is not surprising that this agent is among the most frequently used in the outpatient setting for MSSA infections that require an extended duration of therapy as well as in the inpatient setting for community-acquired bacterial pneumonia (CABP), where MSSA is a commonly isolated pathogen [3]. Since ceftriaxone is frequently utilized in both clinical settings assessing its susceptibility and ultimately the potency of this agent against MSSA is of great importance if optimally effective therapy is to be prescribed.

At the time of this analysis, the FDA breakpoints for ceftriaxone against *S. aureus* were ≤4 μg/mL, 8 μg/mL and ≥16 μg/mL, for susceptible, intermediate, and resistant, respectively assuming the utilization of 2 grams intravenously every 12–24 h [4,5]. This susceptibility breakpoint was reduced based on a reassessment of ceftriaxone’s pharmacodynamic profile showing that the free drug concentration above the MIC (*f*T > MIC) was insufficient to achieve >90% probability of target attainment (PTA) for MSSA with MICs of ≤8 μg/mL [4]. Moreover, this analysis defined the following clinically appropriate susceptibility breakpoints of ≤4 μg/mL with a dose of 2 grams intravenously every 24 h or ≤2 μg/mL with a dose of 1 gram intravenously every 24 h. Additionally, several studies have demonstrated that an overwhelming percentage of MSSA isolates have ceftriaxone MIC of ≥4 μg/mL [6,7]. Overall, these microbiologic and pharmacodynamic data demonstrated the importance of ceftriaxone dose selection and the impact of the MSSA MIC distribution on the achievement of sufficiently high PTAs to ensure good clinical outcomes.

More recently the CLSI eliminated the MIC based breakpoints for many β-lactams for MSSA and now suggests the use of oxacillin or cefoxitin as a surrogate agent for predicting the susceptibility of MSSA to many antimicrobials including ceftriaxone [8,9]. Although the application of this testing algorithm reduces the required testing in the clinical microbiology laboratory, a study that reported the importance of the ceftriaxone MIC in the treatment of MSSA suggested that the use of this surrogate approach is reliable only when the MIC for oxacillin is ≤0.5 μg/mL and the higher dose of 2 grams daily is utilized [6]. In addition to the CLSI action as noted above, the FDA also removed the ceftriaxone susceptibility breakpoints for MSSA from the compounds package insert and instead recommends a daily dose of 2 to 4 grams for MSSA infection [10].

Herein, we aimed to (1) assess the predictive value of surrogate testing of oxacillin and cefoxitin for commonly utilized parenteral antibiotics for MSSA; (2) to evaluate the concordance between predictive susceptibility testing and the *in vivo* exposures for ceftriaxone.

## 2. Methods

MSSA were collected from U.S. medical centers over the period of 2011 to 2013. Isolates were obtained from all anatomical sites with the exception of the urinary tract. Once collected, the organisms were transferred to trypticase soy agar slants for shipment to the central laboratory (Center for Anti-Infective Research and Development, Hartford Hospital, Hartford Hospital, Hartford, CT, USA) for MIC determinations.

MIC testing was conducted for cefazolin, ceftaroline, cefoxitin, ceftriaxone, nafcillin, and oxacillin using broth microdilution according to CLSI recommendations. For quality control purposes, the ATCC *S. aureus* 29,213 was used on all MIC trays prior to and during the susceptibility testing. Results were interpreted based on the current CLSI or FDA breakpoint criteria listed in Table 1. The susceptibility profiles of oxacillin and cefoxitin were compared with each of the other antimicrobials tested and the correlation between susceptibility for each pair was analyzed by scattergram plots and error rates. The following definitions were used for the categorization of errors: very major errors (VMEs) when the percent of cefoxitin or oxacillin-susceptible isolates were resistant to comparators (cefazolin, ceftaroline, ceftriaxone, and/or nafcillin), major errors (MEs) when the percent of cefoxitin or oxacillin-resistant isolates were susceptible to comparators, and minor errors for any categorical disagreements that resulted in an intermediate for comparator and either susceptibility or resistance for cefoxitin or oxacillin. CLSI defines an acceptable interpretive results when the sum of VMEs and MEs is 3% or less and minor errors 10% or less [8].

**Table 1:** Breakpoint criteria for antibiotics used in the analysis.

Classifications	MIC Breakpoints, μg/mL (S/I/R)					
	Cefazolin	Cefoxitin	Ceftaroline	Ceftriaxone	Nafcillin	Oxacillin
FDA	≤16/-/≥32	≤4/-/≥8	≤1/2/≥4	≤4/8/≥16 *	≤2/-/≥4	≤2/-/≥4
CLSI 2013	----	≤4/-/≥8	≤1/2/≥4	----	----	≤2/-/≥4
PD breakpoint	----	----	----	≤2/4/≥8 **	----	----

S: susceptible; I: intermediate; R: resistant; *: FDA recommended dose: 2 gm IV q12–24 h; **: PD breakpoint dose: 1 gm IV q24 h.

Given the multitude of ceftriaxone doses used clinically, a pharmacodynamic breakpoint of 2 μg/mL was used for ceftriaxone 1 gram every 24 h regimen and the 4 μg/mL value was employed when considering the higher 2 gram every 12–24 h dose of ceftriaxone [4].

## 3. Results

A total of 1238 MSSA isolates were collected from 42 U.S. medical centers and were sent to the central laboratory for susceptibility testing. Oxacillin demonstrated a good surrogate predictive value SPV of susceptibility for cefazolin, ceftriaxone, ceftaroline, and nafcillin with excellent agreement between susceptible, intermediate, and resistant categories and only low minor error reported with ceftriaxone. A fourteen percent VME was associated with oxacillin used as a surrogate of susceptibility for cefoxitin. Table 2 describes the error rate for each of the antimicrobials against the surrogate agent. Based on the previous FDA breakpoints of ≤4 μg/mL, only 62 (5%) isolates were ceftriaxone non-susceptible and all of these organisms displayed an MIC equal to 8 μg/mL. Sixteen percent (*n* = 196) of isolates had a ceftriaxone MIC of ≤2 μg/mL and remaining 980 (79%) had a ceftriaxone MIC of 4 μg/mL. According to the FDA breakpoint of ≤4 μg/mL, oxacillin’s SPV for ceftriaxone produced 5% minor error. However, when considering the resulting *in vivo* exposures obtained when administering the 1 gram every 24 h dose and the overall percentage of isolates that had MICs >2 μg/mL, the discordance between the oxacillin SPV and the pharmacodynamic breakpoint for this dose is 84% (Table 2). Conversely, when using the higher ceftriaxone dosing regimen discordance is only observed in 5% of isolates with ceftriaxone MICs of 8 μg/mL.

**Table 2:** Categorical agreement and error rates for oxacillin and cefoxitin according to the FDA breakpoints.

Surrogate Antibiotic	Comparator Antibiotic	Error Rates (%)		
		Very Major	Major	Minor
Oxacillin	Cefoxitin	14	0	0
	Nafcillin	0	0	0
	Ceftriaxone	0	0	5
	Ceftriaxone PD *	0	0	84
	Cefazolin	0	0	0
	Ceftaroline	0	0	0
Cefoxitin	Nafcillin	0	15	0
	Ceftriaxone	0	13	5
	Ceftriaxone PD *	0	2	84
	Cefazolin	0	14	0
	Ceftaroline	0	15	0

*: Ceftriaxone PD = Ceftriaxone pharmacodynamic breakpoint defined as 2 μg/mL.

Cefoxitin as a surrogate of susceptibility for cefazolin, ceftaroline, ceftriaxone and nafcillin was associated with ≥13% ME. When evaluating ceftriaxone, the 161 (13%) isolates that displayed MICs of ≤2 and 4 μg/mL were resistant to cefoxitin, thus major error was observed (Table 2). In contrast, the 62 (5%) isolates with ceftriaxone MICs of 8 μg/mL displayed varied susceptibility to cefoxitin and thus only minor error was noted. As with oxacillin, this minor error noted in the SPV evaluation jumped to a discordance rate of 84% when considering the pharmacodynamic profile resulting from the administration of 1 gram every 24 h ceftriaxone dosing regimen.

## 4. Discussion

The use of ceftriaxone to treat MSSA infections has increased due to its once-daily dosing regimen, absence of dose adjustments in patients with renal dysfunction, and reduced cost [11]. Current CLSI recommendations propose the use of surrogate agents such as oxacillin and cefoxitin to predict the susceptibility of ceftriaxone and other β-lactams against MSSA [8]. In the current study we demonstrated that oxacillin displayed reliable SPVs for cefazolin, ceftaroline, and nafcillin with excellent agreement between susceptible, intermediate, and resistant categories. For ceftriaxone, oxacillin had a reliable SPV with 5% minor error, which is well within CLSI’s acceptable limits when the FDA breakpoint was utilized. Conversely, cefoxitin appears to have a poor SPV against all agents tested as major errors ≥13% were observed with cefazolin, ceftriaxone, ceftaroline and nafcillin. In addition to these major errors, cefoxitin produced an additional 5% minor error against ceftriaxone which further reduces its utility as an SPV for this agent. Moreover, the 14% very major errors observed with oxacillin when considering its surrogacy against cefoxitin appears to explain the discordance of SPV results between the two surrogate agents and their respective predictive utility for the susceptibility of the tested β-lactams against MSSA.

While oxacillin appears to have acceptable SPVs for the β-lactams tested, careful consideration should be taken with the interpretation of these results as this approach predicts the likelihood of susceptibility based on laboratory criteria, but does not describe the reduced *in vivo* potency of ceftriaxone when considering the MIC distribution of MSSA as well as the variety of doses used in clinical practice. To add additional confusion for the clinician utilizing ceftriaxone for MSSA infections, both the FDA and the CLSI have now abandoned the use of ceftriaxone specific susceptibility criteria for MSSA, thus the prescriber has no potency reference on which to base decisions regarding the appropriateness of dose. To further emphasize the clinical challenges associates with the appropriate use of ceftriaxone for MSSA, although the package insert advocates a dose of 2 to 4 gm per day for MSSA infections [10], the 1 gram once-daily dose continues to be a commonly utilized and guideline advocated regimen for the empiric therapy of CABP where MSSA is a frequently isolated pathogen [12,13,14].

In this study, we further highlight the importance of dose selection in order to optimize the pharmacodynamic profile of ceftriaxone for the management of MSSA based infection. As a result of the current MIC distribution of ceftriaxone against this national collection of MSSA, low dosing regimens appear insufficient to drive the most favorable outcomes for this pathogen. To test this hypothesis, Iacovides and colleagues assessed the bactericidal activity of ceftriaxone against MSSA using an *in vitro* pharmacodynamic model [15]. These investigators tested 5 clinical isolates of MSSA with ceftriaxone MICs ranging from 2 to 8 μg/mL. The study revealed a marked reduction in ceftriaxone bactericidal activity over the range of MICs tested, with appreciable bacterial killing at 24 h only observed when the ceftriaxone MIC was 2 μg/mL. While this *in vitro* pharmacodynamic assessment appears to suggest the inadequacy of low dose ceftriaxone regimens for MSSA infection, additional clinical data are required to determine if the poor outcomes are associated with these doses for the management of MSSA in the setting of CABP.

In summary, cefoxitin is frequently associated with major errors and is therefore not a reliable surrogate for the susceptibility of the β-lactams tested. While cefoxitin was a poor surrogate, our data indicate that oxacillin has excellent surrogate predictive value for cefazolin, ceftriaxone, ceftaroline and nafcillin. While oxacillin is an adequate surrogate based on the defined laboratory criteria, our data also suggest that caution should be exercised when utilizing this testing approach because the MIC distribution of MSSA as well as the variety of doses used in clinical practice may result in inadequate *in vivo* exposures of ceftriaxone.
